# Fbxo45 facilitates pancreatic carcinoma progression by targeting USP49 for ubiquitination and degradation

**DOI:** 10.1038/s41419-022-04675-2

**Published:** 2022-03-12

**Authors:** Linhui Wu, Ke Yu, Kai Chen, Xuelian Zhu, Zheng Yang, Qi Wang, Junjie Gao, Yingying Wang, Tong Cao, Hui Xu, Xueshan Pan, Lixia Wang, Jun Xia, Yuyun Li, Zhiwei Peter Wang, Jia Ma

**Affiliations:** 1 Bengbu Medical College Key Laboratory of Cancer Research and Clinical Laboratory Diagnosis, Bengbu Medical College, Bengbu, Anhui 233030 China; 2 Department of Laboratory Medicine, School of Laboratory Medicine, Bengbu Medical College, Bengbu, Anhui 233030 China; 3 Department of Pathology, Bengbu Medical College, Bengbu, Anhui 233030 China; 4 Department of Clinical Laboratory, The First Affiliated Hospital of Bengbu Medical College, Bengbu, Anhui 233004 China; 5 Department of Clinical Laboratory Diagnostics, School of Laboratory Medicine, Bengbu Medical College, Bengbu, Anhui 233030 China; 6 Department of Biochemistry and Molecular Biology, School of Laboratory Medicine, Bengbu Medical College, Bengbu, Anhui 233030 China; 7 Department of Urology, The Second Affiliated Hospital of Soochow University, Suzhou, Jiangsu 215006 China; 8 Center of Scientific Research, the Second Affiliated Hospital of Wenzhou Medical University, Wenzhou, Zhejiang 325027 China

**Keywords:** Pancreatic cancer, Pancreatic cancer

## Abstract

Fbxo45, a conserved F-box protein, comprises of an atypical SKP1, CUL1, F-box protein (SCF) ubiquitin ligase complex that promotes tumorigenesis and development. However, the biological function and molecular mechanisms of Fbxo45 involved in pancreatic carcinogenesis are ambiguous. We conducted several approaches, including transfection, coIP, real-time polymerase chain reaction (RT-PCR), Western blotting, ubiquitin assays, and animal studies, to explore the role of Fbxo45 in pancreatic cancer. Here, we report that USP49 stability is governed by Fbxo45-mediated ubiquitination and is enhanced by the absence of Fbxo45. Moreover, Fbxo45 binds to a short consensus sequence of USP49 through its SPRY domain. Furthermore, Fbxo45-mediated USP49 ubiquitination and degradation are enhanced by NEK6 kinase. Functionally, Fbxo45 increases cell viability and motility capacity by targeting USP49 in pancreatic cancer cells. Xenograft mouse experiments demonstrated that ectopic expression of Fbxo45 enhanced tumor growth in mice and that USP49 overexpression inhibited tumor growth in vivo. Notably, Fbxo45 expression was negatively associated with USP49 expression in pancreatic cancer tissues. Fbxo45 serves as an oncoprotein to facilitate pancreatic oncogenesis by regulating the stability of the tumor suppressor USP49 in pancreatic cancer.

## Introduction

Pancreatic cancer (PC) is a common malignant digestive tract tumor with an increasing incidence rate and death rate over the past decade [1]. PC patients often have a poor survival because the 5-year relative survival rate for PC is 9% [1]. Approximately 60,430 new cases and 48,220 deaths of PC will be counted in the United States in 2021 [1]. There were 458,918 cases and 432,242 deaths of PC worldwide in 2018 [2]. Only ~20% of PC patients can be treated by surgical excision, and the remaining 80% of PC patients have a poor prognosis due to distant metastasis and locally advanced stages [3]. In addition to the high aggressiveness of PC, the poor survival rate might be due to chemotherapeutic resistance and a lack of effective therapeutic targets [3]. Several genetic variations are distinctly related to pancreatic carcinogenesis, such as *KRAS*, *BRCA1*, and *BRCA2* mutations and *Wnt* and *Notch* mutations [4–6]. Targeted therapy has not yielded impressive outcomes for PC patients. Thus, exploration of the molecular mechanism of PC and development of new therapeutic targets and agents are urgent.

A canonical SKP1-CUL1-F-box protein (SCF) complex is composed of four subunits, including an adaptor protein SKP1, a scaffold protein Cul1, a ring finger protein RBX1 to bind with E2-conjugating enzyme and an F-box protein to recognize the specific substrate [7–9]. Fbxo45 is a conserved F-box protein that constitutes an SCF E3 ligase complex that controls protein degradation [10]. Fbxo45 forms an atypical SCF complex that contains Fbxo45, SKP1, and PAM (also named MYCBP2) but lacks Cul1 [11]. A few substrates of Fbxo45 have been reported, including p73, Par-4, and FBXW7, all of which account for regulating cell survival, cell apoptosis, neural development, and carcinogenesis [12–17]. Fbxo45 was recently identified to promote tumor initiation and progression, suggesting that the Fbxo45 oncoprotein might be a potential tumor therapeutic target [18].

Ubiquitin-specific peptidase 49 (USP49) is a member of the deubiquitinating enzyme (DUB) family, which participates in the removal of ubiquitin and enhances its targeting protein stability. USP49 has been demonstrated to deubiquitinate H2B and mediate cotranscriptional splicing of abundant exons [19]. Furthermore, USP49 binds to and deubiquitinates MITA, thus reducing antiviral responses after HSV-1 infection [20]. USP49 has been reported to deubiquitinate DUSP1, which mediates the DUSP1-JNK signaling pathway and plays a protective role in cardiac ischemia/reperfusion (I/R) injury [21]. USP49 mediates AG3 ubiquitination levels and subsequently regulates the anti-HIV-1 activity [22]. Moreover, USP49 deubiquitinates and stabilizes FKBP51, consequently inhibiting the FKBP51-PHLPP-AKT pathway and suppressing tumorigenesis and chemoresponse in PC [23]. Consistently, USP49 also exerts a tumor suppressive function in colon cancer and modulates the P53-mediated DNA damage response (DDR) by upregulating P53 expression, which in turn enhances USP49 expression to form a positive feedback loop [24]. Here, we report that Fbxo45 has a carcinogenic function in PC through an underlying molecular mechanism via interaction with and ubiquitin-mediated degradation of USP49. These results may provide a potential therapeutic strategy for PC patients.

## Results

### Fbxo45 negatively regulates USP49 stability through ubiquitinated degradation

**Fig. 1:**
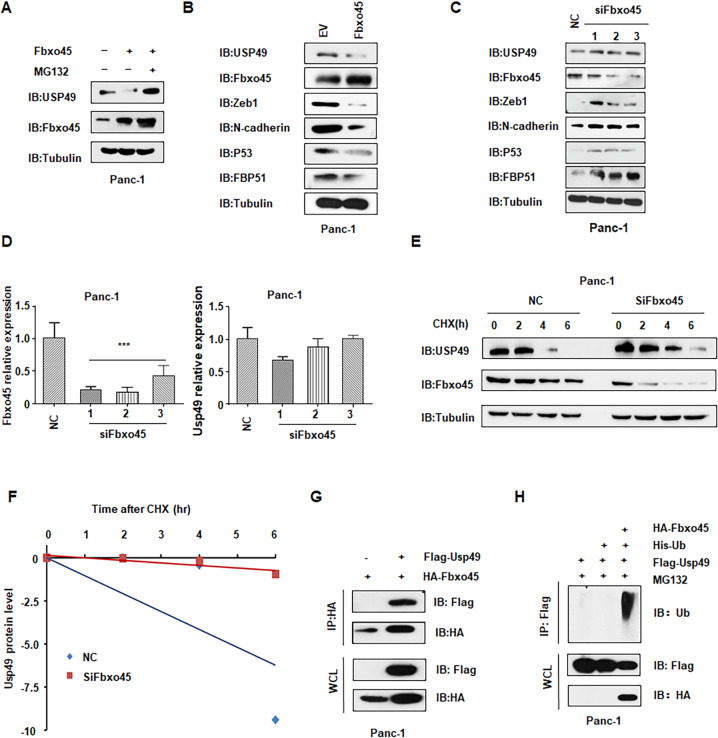
Fbxo45 negatively regulates USP49 stability through ubiquitinated degradation. **A** IB analysis of whole-cell lysates (WCLs) derived from Panc-1 cells transfected with the indicated plasmids, which were treated with 10 μM MG132 for 6 h before harvesting. **B** IB analysis of WCLs derived from Panc-1 cells transfected with Fbxo45 constructs or EV. EV: empty vector. **C** IB analysis of WCLs derived from Panc-1 cells transfected with Fbxo45 siRNA or the negative control (NC). **D** qRT-PCR analysis to detect Fbxo45 and USP49 mRNA levels after Fbxo45 depletion in 293 T cells. Data are shown as the mean ± SD of three independent experiments. ***p < 0.001 compared to NC. **E** IB analysis of WCLs derived from Panc-1 cells after the specified duration of 100 μg/ml cycloheximide (CHX) transfection with Fbxo45 siRNA. **F** USP49 protein abundance in (E) was quantified and plotted. **G** IB analysis of immunoprecipitates (IPs) and WCLs derived from Panc-1 cells transfected with the indicated plasmids. Cells were treated with 10 μM MG132 for 6 h before harvesting. **H** IB analysis of ubiquitination products and WCLs derived from Panc-1 cells transfected with the indicated constructs. Cells were treated with 10 μM MG132 for 6 h before harvesting.

To understand whether *USP49* expression was regulated by *Fbxo45* through ubiquitinated degradation, we measured the *USP49* expression levels in multiple cell lines after *Fbxo45* modulation. We observed the basal expression levels of Fbxo45 and USP49 in several pancreatic cancer cell lines (Supplementary Fig. 1A, B). We found that ectopic overexpression of *Fbxo45* significantly downregulated the abundance of USP49 in 293 T cells and Panc-1 and PaTu-8988 cells, but Fbxo45-mediated USP49 downregulation was blocked by pretreatment with the proteasome inhibitor MG132 in 293 T cells and Panc-1 cells (Fig. 1A, B, Supplementary Fig. 1C, D). Consistently, we also observed that USP49 protein expression was upregulated after *Fbxo45* knockdown by siRNA treatment in multiple cell lines (Fig. 1C, Supplementary Fig. 1E). In addition, the expression of downstream targets of Fbxo45, including Zeb1 and N-cadherin, was also changed in multiple cell lines after *Fbxo45* modulation [25] (Fig. 1C, Supplementary Fig. 1E). The expression of p53 and FKBP51, two downstream factors of USP49 [23, 24], was also affected by *USP49* modulation (Fig. 1C, Supplementary Fig. 1E). Importantly, the mRNA levels of *USP49* were barely changed by *Fbxo45* siRNA transfection in multiple cell lines (Fig. 1D and Supplementary Fig. 1F–H). Consistent with these results, the half-life of USP49 was prolonged after knockdown of endogenous *Fbxo45* (Fig. 1E, F and Supplementary Fig. 2A–D), suggesting that Fbxo45 controls USP49 expression primarily through mechanistic posttranslational modification. Moreover, coIP assays showed that Fbxo45 specifically interacted with USP49 in Panc-1 cells and 293 T cells (Fig. 1G and Supplementary Fig. S2E, F). Furthermore, our results also demonstrated that Fbxo45 promoted USP49 ubiquitination (Fig. 1H and Supplementary Fig. 2G). Therefore, all of the above results indicated that Fbxo45 could decrease USP49 abundance through ubiquitinated degradation.

### Fbxo45-induced USP49 ubiquitination and degradation depend on its SPRY domain

**Fig. 2:**
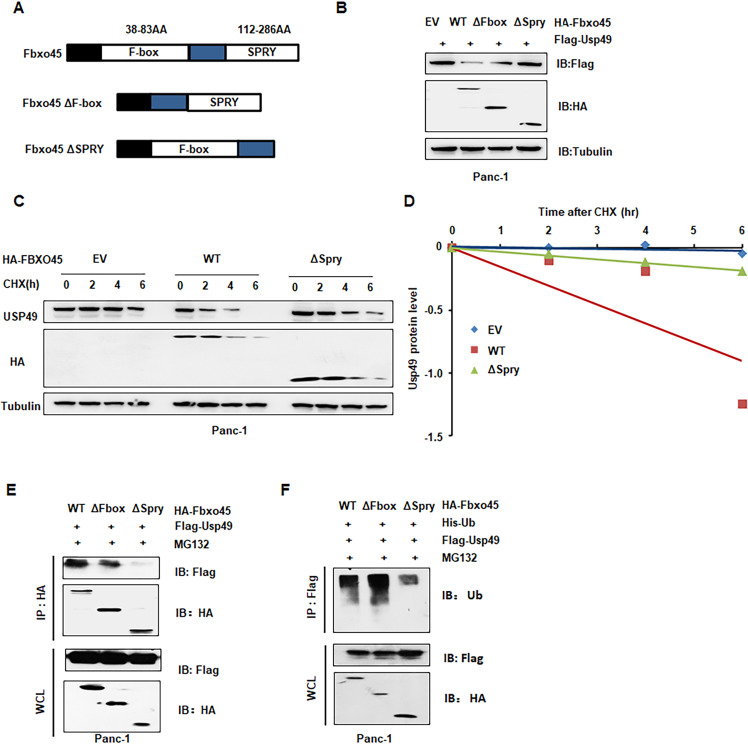
Fbxo45-induced ubiquitination and degradation of USP49 depends on its SPRY domain. **A** Schematic of Fbxo45 domains and its mutant construct. **B** IB analysis of WCLs derived from Panc-1 cells transfected with the indicated plasmids. **C** IB analysis of WCLs derived from Panc-1 cells after the specified duration of 100 μg/ml cycloheximide (CHX) transfection with the indicated constructs. **D** USP49 protein abundance in (**C**) was quantified and plotted. **E** IB analysis of IPs and WCLs derived from Panc-1 cells transfected with the indicated plasmids. Cells were treated with 10 μM MG132 for 6 h before harvesting. **F** IB analysis of ubiquitination products and WCLs derived from Panc-1 cells transfected with the indicated constructs. Cells were treated with 10 μM MG132 for 6 h before harvesting.

Fbxo45 contains two conserved domains, including an F-box domain functionally interacting with endogenous Skp1 to form the SCF complex and an SPRY domain responsible for binding to substrates [12, 13]. To determine whether Fbxo45 mediated the degradation of USP49 through its SPRY domain, we constructed Fbxo45 ΔF-box and Fbxo45 ΔSPRY mutants (Fig. 2A). As shown in our IB results, ΔF-box and ΔSPRY mutants, especially ΔSPRY mutants, largely blocked Fbxo45-mediated degradation of USP49 (Fig. 2B and Supplementary Fig. 3A). Consistently, Panc-1 cells transfected with the ΔSPRY mutant exhibited an extended half-life of USP49 compared to *Fbxo45* WT and EV transfection (Fig. 2C, D). Moreover, deletion of SPRY dramatically attenuated the interaction of USP49 compared with deletion of the F-box and WT of *Fbxo45* in 293 T cells and Panc-1 cells (Fig. 2E, Supplementary Fig. 3B). Furthermore, deletion of SPRY abrogated the ubiquitination of USP49 compared with the F-box deletion and WT of *Fbxo45* in Panc-1 cells and 293 T cells (Fig. 2F, Supplementary Fig. 3C). These results indicated that the SPRY domain was responsible for the interaction with and ubiquitination degradation of USP49.

### Fbxo45 binds to a conserved motif in USP49

**Fig. 3:**
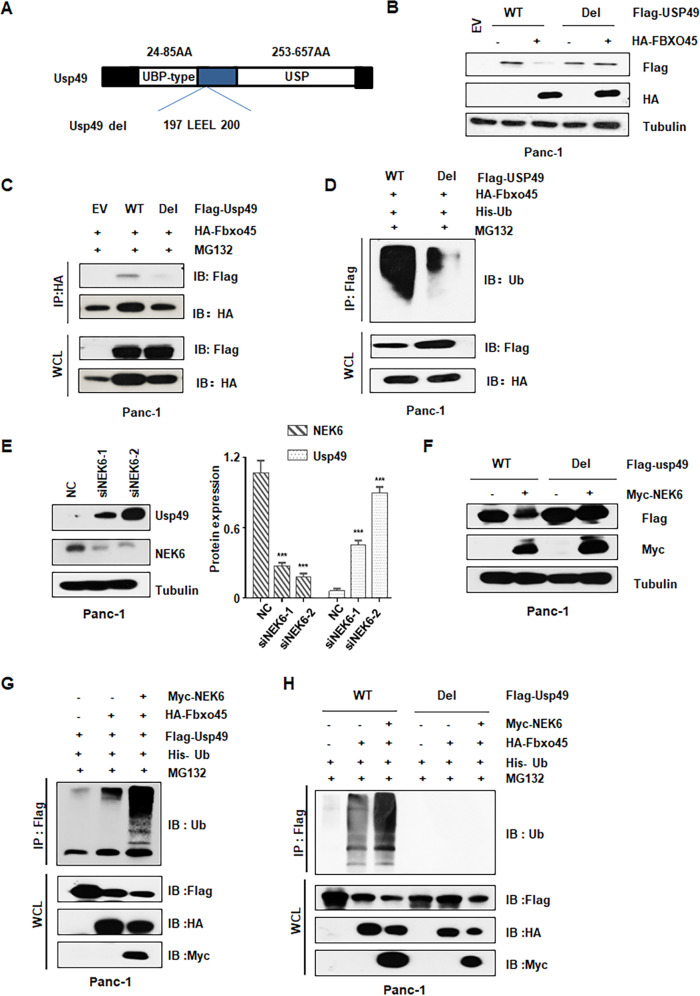
Fbxo45 binds to a conserved motif in USP49 and NEK6 enhances the degradation of USP49. **A** Schematic of USP49 domains and its mutant construct. **B** IB analysis of WCLs derived from Panc-1 cells transfected with the indicated plasmids. **C** IB analysis of IPs and WCLs derived from Panc-1 cells transfected with the indicated constructs. Cells were treated with 10 μM MG132 for 6 h before harvesting. **D** IB analysis of ubiquitination products and WCLs derived from Panc-1 cells transfected with the indicated constructs. Cells were treated with 10 μM MG132 for 6 h before harvesting. **E** IB analysis of WCLs derived from Panc-1 cells transfected with NEK6 siRNAs. **F** IB analysis of WCLs derived from Panc-1 cells transfected with the indicated constructs. **G** IB analysis of ubiquitination products and WCLs derived from Panc-1 cells transfected with the indicated constructs. Cells were treated with 10 μM MG132 for 6 h before harvesting. **H** IB analysis of ubiquitination products and WCLs derived from Panc-1 cells transfected with the indicated constructs. Cells were treated with 10 μM MG132 for 6 h before harvesting.

Having demonstrated Fbxo45 as an upstream E3 ligase of USP49, we further determined the binding sites within USP49 to specifically interact with Fbxo45. Fbxw7a, a target of Fbxo45, has been demonstrated to have a specific interaction domain binding to Fbxo45, which contains conserved acidic amino acid residues [14]. Therefore, we generated a truncated version of USP49 containing the 197-200 AA LEEL deletion (named as Usp49 del, Fig. 3A). We found that *USP49* del blocked Fbxo45-mediated USP49 downregulation (Fig. 3B and Supplementary Fig. 3D). Furthermore, in support of the identified sites of USP49 contributing a critical role in Fbxo45-mediated USP49 degradation, the half-life of USP49 del was prolonged compared with *USP49* WT (Supplementary Fig. 3E, F). More importantly, compared with WT, *USP49* del significantly reduced the USP49 interaction with Fbxo45 (Fig. 3C and Supplementary Fig. 3G). Moreover, ubiquitination of USP49 was dramatically inhibited in USP49 del but not in WT (Fig. 3D and Supplementary Fig. 3H).

### NEK6 enhances the interaction and degradation of USP49 by Fbxo45

Even though substrate phosphorylation catalyzed by a kinase is necessary for ubiquitination and degradation of substrates by many SCF E3 ligases, it was unclear whether the atypical Fbxo45 SCF complex also requires this similar modification for substrate degradation. To identify specific kinase(s) required for USP49 degradation, we used the Scansite program to predict the proper kinase(s) responsible for phosphorylation of USP49. Some NEKs, including NEK1, NEK2, NEK4, and NEK5, were observed to be involved in USP49 Ser/Thr residue phosphorylation by the Scansite program. Interestingly, we observed that only NEK3 and NEK6, but not other NEK family members, could decrease USP49 protein expression under ectopic expression conditions (Supplementary Fig. 4A). However, we found that NEK6 specifically interacted with USP49 but not NEK3, suggesting that NEK6 was critically involved in USP49 degradation (Supplementary Fig. 4B). Consistent with these findings, the deletion of NEK6 by siRNA dramatically enhanced the USP49 abundance (Fig. 3E and Supplementary Fig. 4C). Notably, NEK6 promoted USP49 WT, but not USP49 del degradation under ectopic expression (Fig. 3F and Supplementary Fig. 4D). More importantly, NEK6 strengthened the Fbxo45-mediated ubiquitination of *USP49* WT, while NEK6 had no influence on the ubiquitination of *USP49* del (Fig. 3G, H and Supplementary Fig. 4E). These results indicated that NEK6 has a potential role in the promotion of Fbxo45-mediated ubiquitination and degradation of USP49.

### Fbxo45 negatively regulates USP49-mediated cell proliferation, migration, and invasion

**Fig. 4:**
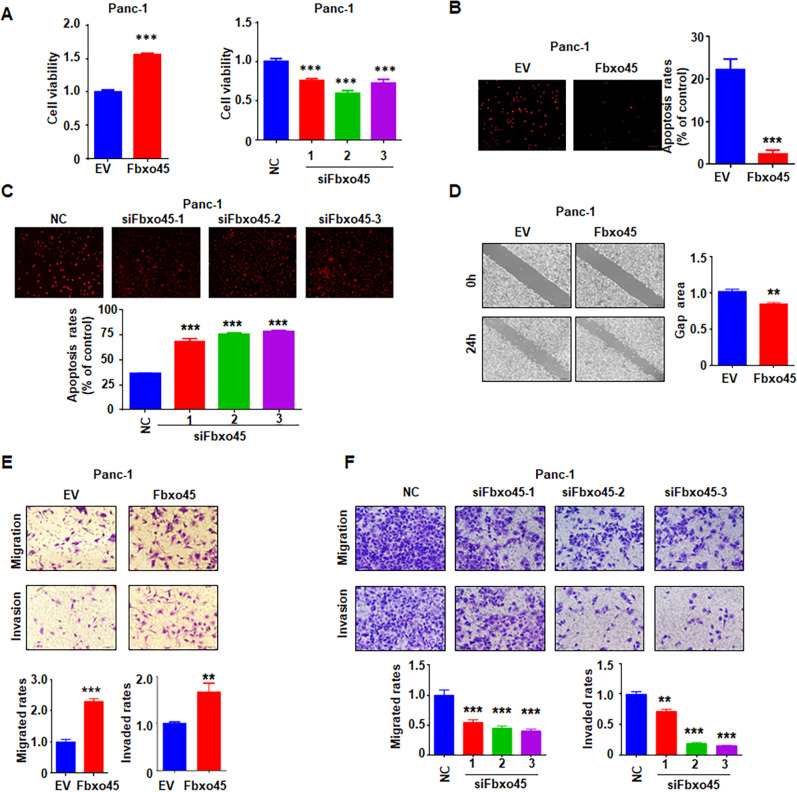
Fbxo45 promotes cell growth, migration, and invasion in PC cells. **A** MTT assays to detect cell proliferation in Panc-1 cells transfected with Fbxo45 siRNAs (Right) or Fbxo45 constructs (Left). Data are shown as mean ± SD of three independent experiments. ***p < 0.001 compared to control. **B**, **C** Tunel assays to detect cell apoptosis of Panc-1 cells transfected with Fbxo45 constructs (**B**) or Fbxo45 siRNAs (**C**). ***p < 0.001 compared to control. **D** Wound healing assays to analyze cell migration capacity of Panc-1 cells transfected with Fbxo45 constructs. **p < 0.01 compared to control. **E** Transwell assays to analyze cell migration and invasion capacity of Panc-1 cells transfected with Fbxo45 cDNA. **p < 0.01 compared to control, ***p < 0.001 compared to control. **F** Transwell assays to analyze cell migration and invasion capacity of Panc-1 cells transfected Fbxo45 siRNAs. **p < 0.01 compared to control, ***p < 0.001 compared to control.

**Fig. 5:**
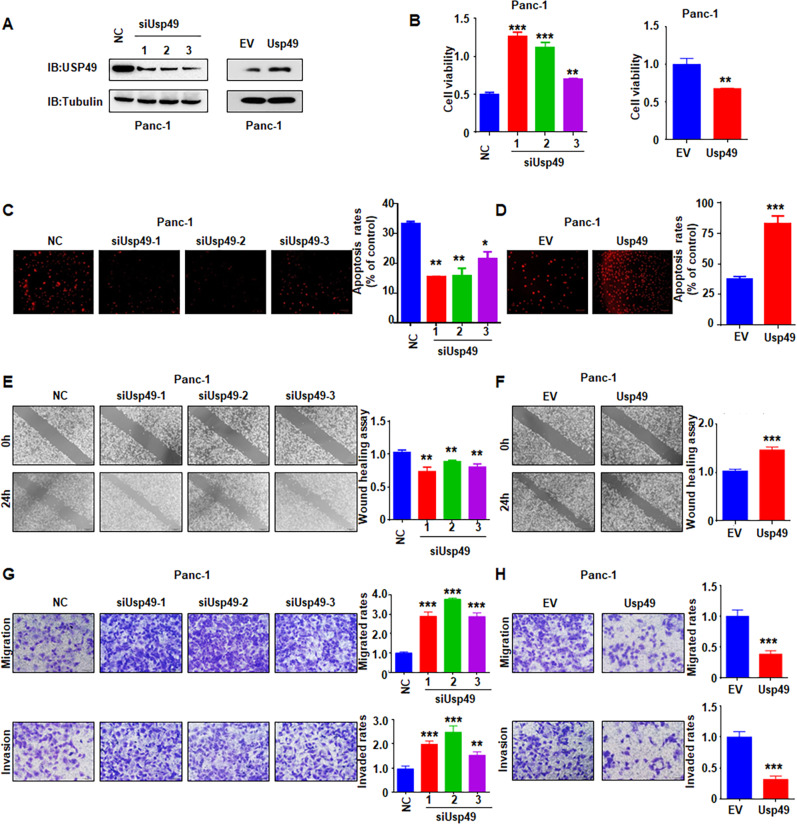
USP49 inhibits cell growth, migration, and invasion in PC cells. **A** IB analysis of WCLs derived from Panc-1 cells transfected with the indicated plasmids. **B** MTT assays to detect cell proliferation of Panc-1 cells transfected with USP49 siRNAs (left) or USP49 cDNA (right). Data were shown as mean ± SD of three independent experiments. **p < 0.01 compared to control, ***p < 0.001 compared to control. **C**, **D** Tunel assays to detect apoptosis of Panc-1 cells transfected with USP49 siRNAs (**C**) or USP49 cDNA (**D**). *p < 0.05, **p < 0.01, ***p < 0.001 compared to control. **E**, **F** Wound healing assays to analyze cell migration capacity of Panc-1 cells transfected with USP49 siRNAs (**E**) and USP49 constructs (**F**). **p < 0.01 compared to control. ***p < 0.001 compared to control. **G**, **H** Transwell assays to analyze cell migration and invasion capacity of Panc-1 cells transfected with USP49 siRNAs (**G**) and USP49 cDNA (**H**). **p < 0.01 compared to control, ***p < 0.001 compared to control.

Although Fbxo45 has been reported to promote carcinogenesis, the biological functions of Fbxo45 in PC are still unclear. To this end, we investigated the effects of Fbxo45 on cell proliferation, apoptosis, migration, and invasion in PC. Ectopic expression of *Fbxo45* promoted cell viability and inhibited cell apoptosis, while deletion of *Fbxo45* suppressed cell viability and induced cell apoptosis in PC at 72 h (Fig. 4A–C, Supplementary Fig. 5A–C). Fbxo45 modification did not change the cell proliferation and apoptosis in PC cells at 24 h (data not shown). Cell migration and invasion capacities were enhanced by *Fbxo45* overexpression but were decreased by *Fbxo45* knockdown in PC at 24 h (Fig. 4D–F, Supplementary Fig. 5D, E). One study has shown that USP49 suppressed PC cell proliferation [23]. Consistently, we found that ectopic expression of *USP49* inhibited PC cell proliferation and induced cell apoptosis, whereas deletion of *USP49* promoted cell viability in PC cells (Fig. 5A–D, Supplementary Fig. 5F–I). Moreover, increased USP49 expression inhibited PC cell migration and invasion, while the decreased expression of USP49 promoted PC cell motility (Fig. 5E–H).

**Fig. 6:**
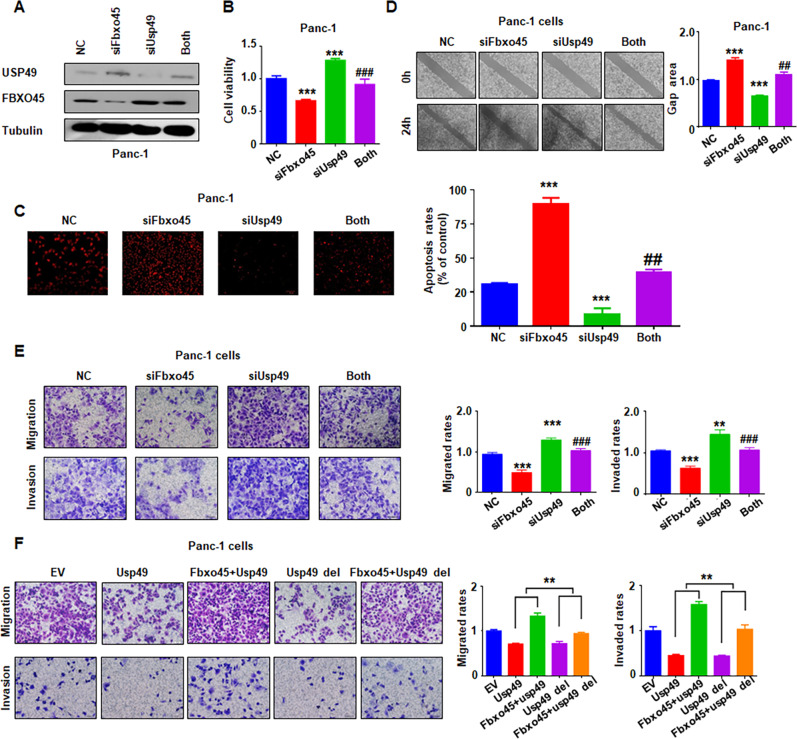
Fbxo45 negatively regulates USP49-mediated cell proliferation, migration, and invasion. **A** IB analysis of WCLs derived from Panc-1 cells transfected with the indicated plasmids. **B** MTT assays to detect the proliferation of Panc-1 cells transfected with the indicated siRNAs and cDNA plasmid. Data are shown as the mean ± SD of three independent experiments. ***p < 0.001 compared to control, ^###^*p* < 0.001 compared to Fbxo45 siRNA-2 alone or USP49 siRNA-2 alone. **C** Terminal deoxynucleotidyl transferase dUTP nick end labeling (Tunel) assays to detect cell apoptosis of Panc-1 cells transfected with the indicated siRNAs. ***p < 0.001 compared to the control, ^##^*p* < 0.01 compared to Fbxo45 siRNA-2 alone or USP49 siRNA-2 alone. **D** Wound healing assays to analyze the cell migratory capacity of Panc-1 cells transfected with the indicated siRNAs. ***p < 0.001 compared to control, ^##^*p* < 0.01 compared to Fbxo45 siRNA-2 alone or USP49 siRNA-2 alone. **E** Transwell assays to analyze the cell migration and invasion capacity of Panc-1 cells transfected with the indicated siRNAs. **p < 0.01, ***p < 0.001 compared to control, ^###^*p* < 0.001 compared to Fbxo45 siRNA-2 alone or USP49 siRNA-2 alone. **F** Transwell assays to analyze the migration and invasion capacity of Panc-1 cells transfected with the indicated plasmids. **p < 0.01 compared to indicated group.

To further dissect the biological functions of Fbxo45 by targeting USP49 degradation, we further detected the influences of simultaneous depletion of Fbxo45 and USP49 on cell proliferation and mobility. PC cells were cotransfected with si*Fbxo45* and si*USP49*, and the protein abundances of USP49 and Fbxo45 were detected by IB to verify simultaneous knockdown (Fig. 6A and Supplementary Fig. 6A). Rescue experiments demonstrated that simultaneous depletion of *Fbxo45* and *USP49* enhanced cell viability compared with *Fbxo45* single deletion and suppressed cell viability compared with *USP49* single knockdown (Fig. 6B and Supplementary Fig. 6B). Consistently, *USP49* knockdown partly annulled *Fbxo45* deletion-induced cell apoptosis (Fig. 6C and Supplementary Fig. 6C). Moreover, USP49 downregulation partly abolished Fbxo45 downregulation-mediated inhibition of cell migration by the wound healing assays and Transwell migration assays (Fig. 6D, E, Supplementary Fig. 6D, E). With the similar results in the Transwell cell invasion assay, si*USP49* treatment was demonstrated to also partly rescued si*Fbxo45* treatment-induced cell invasion inhibition (Fig. 6E, Supplementary Fig. 6D, E). Furthermore, in keeping with the role of the identified motif in mediating Fbxo45-dependent degradation of USP49, coexpression of Fbxo45 was observed to promote *USP49* WT- but not *USP49* del-mediated inhibition of cell migration and invasion (Fig. 6F). Together, these results suggested that Fbxo45 functions as an oncoprotein in PC by targeting the tumor suppressor USP49 for ubiquitination and degradation.

### Fbxo45 promotes PC tumorigenesis

**Fig. 7:**
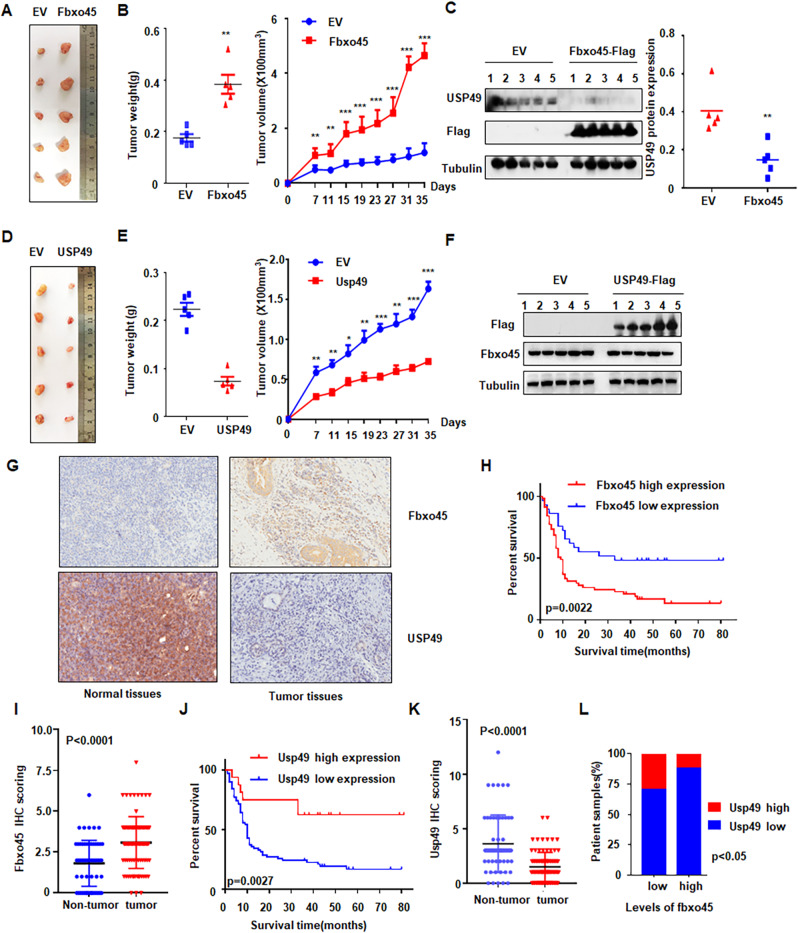
Fbxo45 promotes PC tumorigenesis. **A** Pictures of tumor masses dissected from Fbxo45-overexpressing xenograft mouse models. Panc-1 cells with stable Fbxo45 overexpression and the control cells were injected subcutaneously into the BALB/c-nu/nu mice to establish xenograft mouse models. **B** Tumor weights and tumor volumes of dissected tumor mass in (**A**). **C** IB analysis of the USP49 protein levels in the dissected tumors (left). USP49 protein abundance was quantified and plotted (right). **D** Pictures of tumor masses dissected from USP49-overexpressing xenograft mouse models. Panc-1 cells with stable USP49 overexpression and the control cells were injected subcutaneously into the BALB/c-nu/nu mice to establish xenograft mouse models. **E** Tumor weights and tumor volumes of dissected tumor mass in (**D**). **F** IB analysis of the USP49 protein levels in the dissected tumors. **G** Representative images of Fbxo45 and USP49 IHC staining from pancreatic cancer patients. **H** Survival curve for Fbxo45 in pancreatic cancer patients. **I** IHC scores of Fbxo45 in pancreatic cancer tissues and adjacent normal tissues. **J** Survival curve for USP49 in pancreatic cancer patients. **K** IHC scores of USP49 in pancreatic cancer tissues and adjacent normal tissues. **L** Statistical analyses of the correlation between Fbxo45 and USP49 expression in pancreatic cancer patients.

From in vivo experiments, we observed that overexpression of Fbxo45 in Panc-1 cells promoted tumor growth in xenograft mouse models (Fig. 7A, B). Tumor mass weights and volumes were markedly increased in *Fbxo45*-overexpressing compared with control mice (Fig. 7B). Next, we also performed IB of USP49 on the xenografted tumors, and USP49 expression was demonstrated to be downregulated in *Fbxo45*-overexpressing xenografted tumors (Fig. 7C). Notably, overexpression of USP49 in Panc-1 cells dramatically inhibited tumor growth in mice, displaying a reduction in the weights and volumes of tumor mass (Fig. 7D–F). Furthermore, PC samples were collected to examine the clinical relevance between Fbxo45 and USP49 by IHC detection (Fig. 7G). We found that Fbxo45 expression in tumor tissues was higher than the Fbxo45 expression in tumor-adjacent normal tissues and was associated with survival in PC samples (*p* = 0.0022, Fig. 7H, I). The survival periods of PC patients with high Fbxo45 expression were remarkably shorter than the survival periods of PC patients with low Fbxo45 expression in PC patients (Fig. 7H). Furthermore, we used bioinformatics to analyze the expression of Fbxo45 and USP49 in human pancreatic tumor samples. The data are from GTEx (including 88 cases of normal pancreatic tissues) and TGCA (including 4 cases of tumor-adjacent normal tissues and 178 cases of pancreatic cancer tissues). The bioinformatics results also showed higher expression of Fbxo45 and lower expression of USP49 in cancer tissues than in normal tissues (Supplementary Fig. 7A, B).

USP49 expression was also analyzed using immunohistochemical (IHC) staining in the PC samples (Fig. 7G). USP49 showed a lower level in tumor tissues than in tumor-adjacent normal tissues (Fig. 7K), which was consistent with the bioinformatic results as described above. Patients with high expression of USP49 had notably longer survival periods than patients with lower expression of USP49 in patients with PC, suggesting that USP49 expression was associated with better survival in PC patients (Fig. 7J). Moreover, we found that a low level of USP49 was dramatically associated with tumor grade (*p* = 0.0371) (Table 1). Among 60 cases with high expression of Fbxo45 in tumor tissues, 53 cases showed low USP49 expression (*p* = 0.0320, Table 2). Among 79 cases with high expression of Fbxo45 in tumors and adjacent tissues, 59 cases showed low USP49 expression (*p* = 0.0007, Table 3). These results suggest that Fbxo45 expression was negatively associated with USP49 expression to a certain degree in PC samples (Fig. 7L).

**Table 1:** Correlation of clinicopathological features and USP49 expression in pancreatic cancer samples.

		USP49 expression		
Characteristic	N	Low	High	*p*-value
Gender				0.4502
Male	58	49	9	
Female	32	25	7	
Age(years)				0.6729
<60	49	40	9	
≥60	40	34	6	
Tumor size				0.0844
T1	2	2	0	
T2	71	55	16	
T3–T4	16	16	0	
Pathological grades				0.0371
I–II (include II)	57	45	12	
II–III (include III)	32	29	3	
III–IV (include IV)	1	0	1	
Distant metastasis				1.000
Absent	86	72	14	
Present	1	1	0	
Clinical stages				0.1779
1	37	27	10	
2	50	44	6	
3–4	1	1	0

**Table 2:** Correlation of Fbxo45 expression and USP49 expression in pancreatic cancer tissues.

Tumor	usp49 Low	usp49 High	*p*-value
Fbxo45 Low	21	9	0.032
Fbxo45 High	53	7

**Table 3:** Correlation of Fbxo45 expression and USP49 expression in pancreatic cancer and normal tissues.

Tissues	usp49 Low	usp49 High	*p*-value
Fbxo45 Low	34	37	0.0007
Fbxo45 High	59	20	

## Discussion

Accumulating evidence has demonstrated that Fbxo45 exhibits a critical biological function in carcinogenesis and progression, indicating that targeting Fbxo45 may be a potential strategy for cancer therapy [18, 26]. One study revealed that insulin-like growth factor 2 mRNA binding protein (IMP2) was overexpressed in PC tissues, which was correlated with Fbxo45 expression, suggesting that Fbxo45 was highly expressed in PC specimens [27]. However, the role of Fbxo45 in PC is still ambiguous. Here, we provided experimental evidence to demonstrate the oncogenic role of Fbxo45 in PC. Moreover, our study identified the USP49 as a downstream target of Fbxo45 in PC cells. Furthermore, Fbxo45 exerted its oncogenic function partly via promotion of USP49 ubiquitination and degradation.

One group reported that Fbxo45 intercepted p53-dependent cell apoptosis in breast cancer cells by targeting p73, a member of the p53 family, for ubiquitination and degradation [13]. Moreover, Fbxo45 was suggested to promote cell growth and inhibit cell apoptosis in HeLa cells by promoting Par-4 degradation [12, 15]. Fbxo45 was also revealed to impede cell death in mitosis in U2OS cells by binding to and inducing Fbxw7 degradation [14]. Consistent with these findings, our current study demonstrated that *Fbxo45* elevated cell viability and suppressed apoptosis in PC cells, and overexpression of *Fbxo45* facilitated xenograft tumor growth in vivo. Regarding the influence of *Fbxo45* on cell motility, our data showed that *Fbxo45* promoted the migration and invasion of PC cells. EMT, a process characterized by epithelial cells acquiring mesenchymal features, is correlated with tumor initiation, metastasis, and drug resistance [28, 29]. One previous report showed that ZEB1, a significant activator of the EMT process, was ubiquitinated and degraded through FLASH-dependent SIAH1/2 E3 ligases and Fbxo45 E3 ligase [16]. Similarly, *Fbxo45* silencing led to EMT and enhanced cell migration capacity in lung cancer cells [30]. One study also revealed that Fbxo45 targeted the degradation of multiple EMT-inducing transcription factors (EMT-TFs), including Zeb1, Zeb2, Snai1, Snai2, and Twist1 [25]. Moreover, this study identified that the miR-27a/Fbxo45/EMT-TF axis contributed to EMT development and tumor progression [25]. Recently, another study demonstrated that DNAJB9 blocked the tumor metastasis by enhancing Fbxo45-involved degradation of ZEB1 in TNBC cells [31]. Two groups reported that Fbxo45 can bind to and degrade N-cadherin and regulate neuron migration to affect neuronal differentiation and brain development [32, 33]. Consistently, our study also showed that Fbxo45 can regulate the expression of Zeb1 and N-cadherin in pancreatic cancer cells. These studies indicated that Fbxo45 might exert different roles in the regulation of motility in different types of cancers. Without a doubt, it is necessary to further determine the role of Fbxo45 in the regulation of EMT in PC in the future.

The underlying mechanisms of Fbxo45-mediated oncogenic function in PC were elucidated in this study. We observed that Fbxo45 could bind to specific degrons of USP49 through its SPRY domain and enhance USP49 ubiquitination, subsequently leading to USP49 protein destruction in a posttranslational manner. In agreement with these findings, IHC staining data also demonstrated that a negative correlation existed to some extent between Fbxo45 expression and USP49 expression in PC samples. Furthermore, as Fbxo45 is an atypical SCF complex, it does not know whether substrate phosphorylation is also necessary for Fbxo45-mediated ubiquitination and degradation. Our study showed that NEK6, but not other NEKs, could promote ubiquitinated degradation of USP49.

Several studies have revealed that USP49 acts as a tumor suppressor in multiple types of cancers [23, 34]. *USP49* deletion enhanced HCT116 cells more resistant to etoposide-induced DNA damage and extensively accelerated tumorigenesis in colon cancer by forming a positive feedback with p53 [24]. In addition, USP49 stabilized FKBP51 expression, which in turn negatively mediated AKT activation, to inhibit proliferation and increase sensitivity to gemcitabine in PC cells [23]. Similarly, USP49 suppressed PI3K-AKT signaling to inhibit cell growth and induce cell cycle arrest in lung cancer cells [34]. Furthermore, lncRNA *HLNC1* was reported to bind to and destabilize USP49 to enhance hepatocellular carcinoma progression [35]. Consistent with these reports, we demonstrated that *USP49* inhibited cell viability, induced cell apoptosis, and suppressed cell motility in vitro, as well as depressed xenograft tumor growth in vivo. Importantly, USP49 partly rescued the Fbxo45-mediated promotion of cell growth and migration and invasion in PC cells, indicating that Fbxo45 performs its tumor promoter function via degradation of USP49 in PC cells. Fbxo45 degraded USP49 and caused p53 downregulation in pancreatic cancer cells with TP53 mutations, which is required for in-depth investigation. Taken together, our study elucidates a novel mechanism of Fbxo45-driven PC progression, in which Fbxo45 increases ubiquitination and destruction of the USP49 tumor suppressor protein. Thus, targeting Fbxo45 and USP49 may be a potential strategy for PC treatment.

## Materials and methods

### Cell culture, cells transfection, and reagents

HeLa, 293 T, PaTu-8988, and Panc-1 cells were authenticated by STR profiling and tested for mycoplasma contamination. Cells were cultured in Dulbecco’s Modified Eagle Medium (DMEM) medium supplemented with 10% fetal bovine serum and 1% penicillin and streptomycin at 37 °C and 5% CO_2_. Cells were cultured in 6-well plates overnight and transfected using Lipofectamine 3000 for various siRNAs and plasmids according to the manufacturer’s instructions. After 6 h, we changed the medium with fresh DMEM. The cells were collected for further analysis as described in the results sections.

### Plasmids and siRNAs

*Fbxo45* cDNA and *USP49* cDNA were cloned into the pcDNA3.1 expression vector carrying with HA or Flag Tag for transient transfection and into the pCDH lentiviral vector to establish the stable overexpression cells. The *Fbxo45* ΔF-box and ΔSPRY sequences were subcloned into the pcDNA3.1 expression vector. The *USP49* Δplasmid was purchased from Youbio Biological Technology (Hunan, China). The pcDNA3.1 NEK1 to NEK7 plasmids were obtained from YouBio Biological Technology. The siRNAs were purchased from GenePharma Company (Shanghai, China). The siRNA sequences were as follows: *siFbxo45-1*, 5′-CAG AUA GGA GAA AGA AUU CGA TT-3′; *siFbxo45-2*, 5′-CAG ACG TTA CTA TTA TCC CTA TT-3′; and *siFbxo45-3*, 5′-CTG GTG GAC AAT AAT CTA CTA TT-3′. *siUSP49-1*, 5'- CGG GAU CUC UAC GUG UUC UTT-3'; *siUSP49-2*, 5'- CCG AGU UCA AAG CAC AUU UTT-3'; *siUSP49-3*, 5'-GGG UCC AUG UCG UCU UUG ATT-3'; *siNEK6-1*, 5'-GCC UCU UGA AGC AAC UGA ATT-3'; *siNEK6-2*, 5'-CCG AGA AGU UAC GAG AAC UTT-3'.

### Antibodies

Anti-Flag (20543-1-AP; 1:1,000), anti-HA (51064-2-AP; 1:1,000), anti-MYC (16286-1-AP; 1:2,000) and anti-USP49 (18066-1-AP; 1:1,000) antibodies were purchased from Proteintech. Anti-FBXO45 antibody (orb156851; 1:1,000) was purchased from Biorbyt Company. Anti-tubulin antibody (2128 S; 1:1,000) was purchased from Cell Signaling Technology. Peroxidase-conjugated anti-mouse secondary antibody (70-GAM007, 1:5,000) and peroxidase-conjugated anti-rabbit secondary antibody (70-GAR0072; 1:5,000) were purchased from MultiSciences Company. Immunoprecipitation kit-HA tag immunomagnetic beads (TB100028, 50 μl for IP) and immunoprecipitation kit-DYKDDDDK (Flag) tag immunomagnetic beads (TB101274, 50 μl for IP) were purchased from Sino Biological Company.

### RT-PCR analysis

The mRNA levels of *Fbxo45* and *USP49* were measured by quantitative RT-PCR (qRT-PCR) using SYBR green (TAKARA, Dalian, China), and *GAPDH* was employed as the internal control as previously described [36]. Total RNA was extracted by TRIzol reagent and transcribed into cDNA. RT-PCR analysis was carried out in triplicate using Power SYBR Green PCR Master Mix following the manufacturer’s protocol. The data were obtained by the 2^−ΔΔCt^ method. The primers of *Fbxo45*, *USP49*, and *GAPDH* are listed as follows: *Fbxo45*, forward primer, 5′-GAT GAG AAC AGC GAG GTG TG-3′ and reverse primer, 5′-TGA GCA ATG GGG TTT CGA TG-3′; *USP49*, forward primer, 5′-CAT CCC CTT CTC CCA GAG GA-3′ and reverse primer, 5′-ATG TGA CCT GAC TGA GCA GC-3′; *GAPDH*, forward primer, 5′-CAG CCT CAA GAT CAG CA-3′, and reverse primer, 5′-TGT GGT CAT GAG TCC TTC CA-3′.

### Immunoblotting (IB) and immunoprecipitation (IP)

For IP, the cells were lysed with NP40 lysate with protease inhibitor cocktail (Thermo Fisher Scientific, USA). The protein concentration was quantified by the bicinchoninic acid (BCA) method. The following IP process was performed as described previously [37]. Approximately 1–2 mg protein was incubated and immunoprecipitated with HA/Flag Tag magnetic beads, subsequently washed five times with TBST buffer and eluted by boiling in sodium dodecyl sulfate (SDS) loading buffer. For IB, the whole cell lysates were obtained using radio-immunoprecipitation assay (RIPA) buffer containing protease inhibitor cocktail (Thermo Fisher Scientific, USA) on ice. Then, the proteins were separated by SDS-polyacrylamide gel electrophoresis (PAGE), subsequently transferred onto polyvinylidene fluoride (PVDF) membranes, and blocked with 5% nonfat dry milk in tris-buffered saline with Tween (TBST) buffer. Then, IB was conducted by incubation with the antibodies at 4 °C overnight. The membrane was washed three times using TBST buffer and incubated with a secondary antibody for 1 h. Then, the membrane was imaged using enhanced chemiluminescence (ECL) buffer to detect the expression of proteins.

### Ubiquitination assay

The 293 T cells and Panc-1 cells were transfected with His-ubiquitin plasmids and the desired plasmids. Thirty-six hours after transfection, cells were cultured with 10 μM MG132 (Sigma) for 6 h. Then, the cells were harvested and lysed with IP lysis buffer. Cell lysate (1000 μg) were incubated with anti-USP49 antibody for 4 h in a cold room. Then, Protein A/G plus agarose was added overnight, and IB verification was subsequently performed after the beads were removed by washing three times.

### Protein half-life analysis

The 293 T cells and HeLa cells were transfected with the desired constructs. Forty hours after transfection, cells were incubated with 20 μg/ml cycloheximide (Sigma). Then, the cells were harvested at various time points and tested by IB for protein abundance.

### MTT assay

The cells were pretreated with the indicated different plasmids overnight and were subsequently seeded in 96-well plates for 72 h incubation. Then, an MTT assay was applied to measure cell viability as described previously [36]. Briefly, the 5 × 10^3^ transfected cells were seeded in 6-well plates for 72 h. Ten microliters of MTT solution (0.5 mg/ml) was added to plates and incubated for 4 h incubation. After removing the supernatant of plates, 100 μl dimethyl sulfoxide (DMSO) was added. The absorption at 490 nm was detected by a Multimode Reader of SpectraMax M5.

### TUNEL assay

Terminal deoxynucleotidyl transferase dUTP nick end labeling (TUNEL) assay was performed in PC cells after different transfections. Briefly, transfected cells were fixed with 4% paraformaldehyde for 25 min at 4 °C and then permeabilized with proteinase K solution for 10 min. Then, cells were incubated with TUNEL reagent (Roche, Basel, Switzerland) for 1 h at 37 °C and counterstained with 4ʹ, 6-diamidino-2-phenylindole (DAPI) for 10 min. Images were photographed under a fluorescence microscope (Leica, Wetzlar, Germany).

### Wound healing assay

The cells were seeded in 6-well plates after different treatments. When cell confluence was over 90%, a wounded scrape was made by a pipette tip crossing the layer of cells. The floating cells were washed three times with phosphate-buffered saline (PBS). Then, the fresh medium was added and wound healing images were photographed at 0 h and 20 h.

### Transwell migration and invasion assays

Cell migration and invasion capacities were detected by Transwell assays using a Transwell chamber (Corning, USA) as described before [36]. The transfected cells were seeded in the upper chamber with serum-free medium. Complete medium with 10% FBS was added to the lower chamber. After 24 h, the migrated and invaded cells on the chamber’s bottom surfaces were fixed in 4% paraformaldehyde and stained in Giemsa. The stained cells were imaged and counted in five random fields for quantification.

### In Vivo animal experiments

Animal experiments were performed as described before [38]. Panc-1 cells with stable overexpression of Fbxo45 or USP49 were inoculated subcutaneously into the flanks of nude mice (5 mice/group) at 5 weeks old. The mice were inspected to measure the tumor sizes every 4 days. The tumor volume was calculated by *L* × *W*^2^ × 0.52 (*L*: the longest diameter; *W*: the shortest diameter). Tumors were excised after 4 weeks of injections and tumors were resected. All animal studies were approved by the Animal Experimentation Ethical Committee of Bengbu Medical College (Bengbu, Anhui, China).

### Human pancreatic tumor samples

Samples of human pancreatic cancer tissues and normal tissues were obtained from OUTDO BIOTECH (Shanghai, China). The expression of Fbxo45 and USP49 was detected by IHC analysis in the same samples as described before [37]. The slides were cooled to room temperature, and 3% H_2_O_2_ was added for 10 min. Then, after slides were incubated with bovine serum, the slides were incubated with anti-Fbxo45 and anti-USP49 antibodies in a cold room overnight. After slides were incubated with streptavidin-conjugated horseradish peroxide for 1 h, slides were treated with 3,3'-diaminobenzidine (DAB) for 5 min. Images were obtained by the camera and analyzed by two independent pathologists.

### Statistical analyses

For MTT, wound healing assay, Transwell assay, qRT-PCR, and tumor size, the significance was analyzed by two-tailed Student’s *t*-test for two groups and ANOVA test for multiple groups. Chi‑squared or Fisher’s exact tests were used to analyze Fbxo45 and USP49 expression in human samples. *p* < 0.05 was considered statistically significant.

## Supplementary information

Supplementary figures

aj-checklist

Editing certificate

## Data Availability

The datasets generated in the current study are available from the corresponding author on reasonable request.
