# Supplementary material for: Fbxo45 facilitates pancreatic carcinoma progression by targeting USP49 for ubiquitination and degradation
Source: Cell Death Dis. 2022 Mar 12;13(3):231. doi: 10.1038/s41419-022-04675-2 (PMC8918322; doi:10.1038/s41419-022-04675-2)
Supplement: Supplementary file 1 — Supplementary figures [file 41419_2022_4675_MOESM1_ESM.pdf]

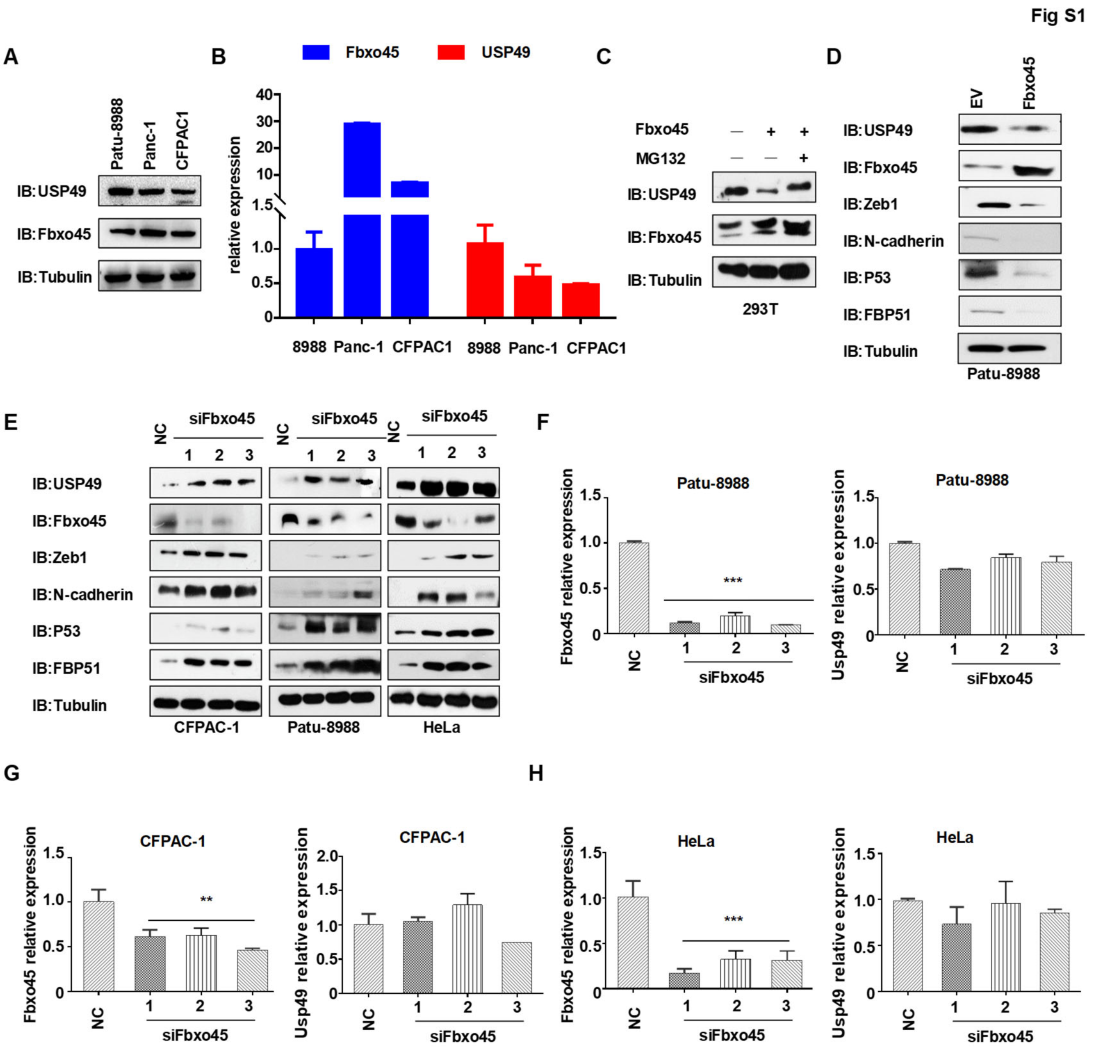

Figure S1: Fbxo45 negatively regulates USP49 stability.

A. IB analysis of whole cell lysates (WCLs) derived from pancreatic cancer cell lines.

B. Quantitative data are presented for Panel A.

C. IB analysis of WCLs derived from HEK293T cells transfected with the indicated plasmids, which were treated with 10 μM MG132 for 6 hours before harvesting.

D. IB analysis of WCLs derived from Patu-8988 cells transfected with Fbxo45 constructs or EV. EV: empty vector.

E. IB analysis of WCLs derived from cancer cells transfected with Fbxo45 siRNA or the negative control (NC).

F-H. qRT-PCR analysis to detect Fbxo45 and USP49 mRNA levels after Fbxo45 depletion in Patu-899 (F), CFPAC-1 (G) and HeLa cells (H). Data are shown as the mean ± SD of three independent experiments. $**p<0.01$, $***p<0.001$ compared to NC.

Fig S2

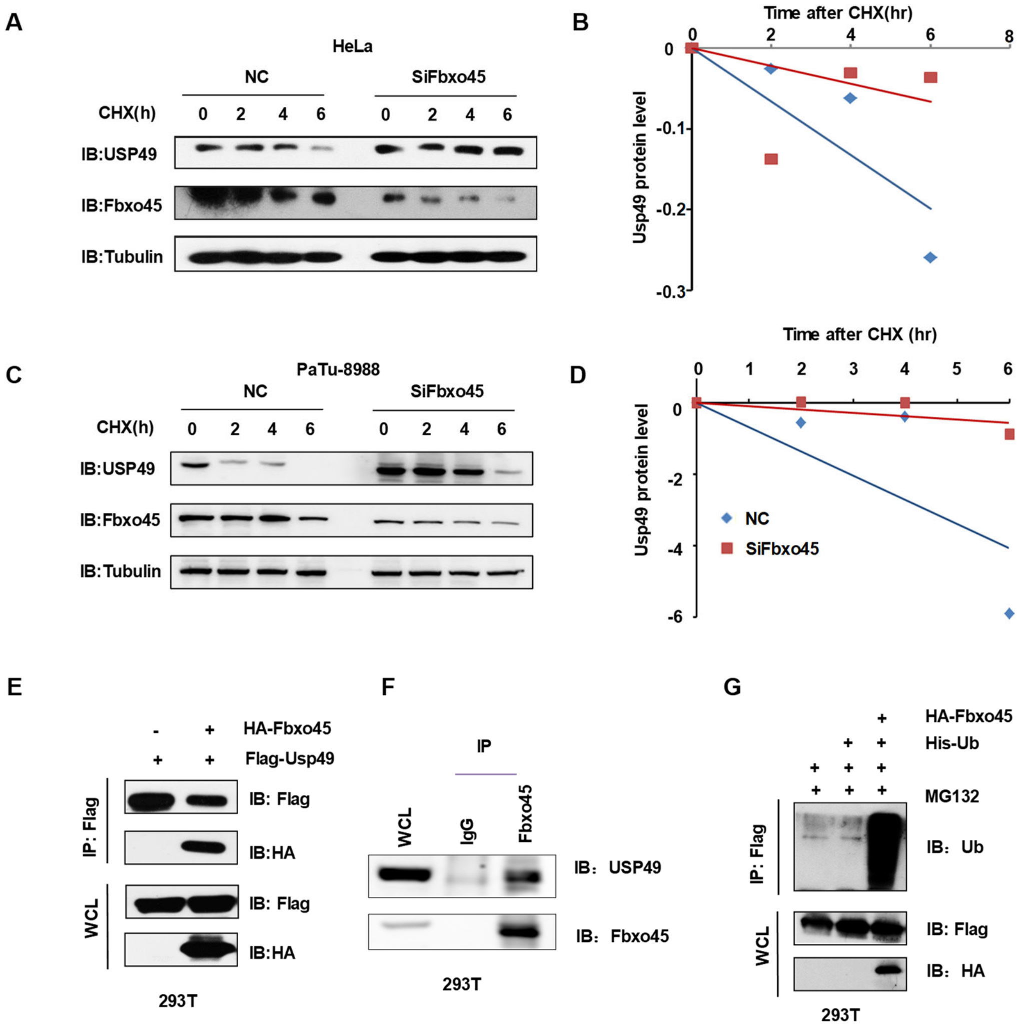

Figure S2: Fbxo45-induced ubiquitination and degradation of USP49.

A. IB analysis of WCLs derived from HeLa cells after the specified duration of 100 μg/ml cycloheximide (CHX) transfection with the indicated constructs.

B. USP49 protein abundance in (A) was quantified and plotted.

C. IB analysis of WCLs derived from PaTu-8988 cells after the specified duration of 100 μg/ml cycloheximide (CHX) transfection with the indicated constructs.

D. USP49 protein abundance in (C) was quantified and plotted.

E. IB analysis of immunoprecipitates (IPs) and WCLs derived from 293T cells transfected with the indicated plasmids. Cells were treated with 10 μM MG132 for 6 hours before harvesting.

F. IB analysis of immunoprecipitates (IPs) and WCLs derived from 293T cells. Cells were treated with 10 μM MG132 for 6 hours before harvesting.

G. IB analysis of ubiquitination products and WCLs derived from 293T cells transfected with the indicated constructs. Cells were treated with 10 μM MG132 for 6 hours before harvesting.

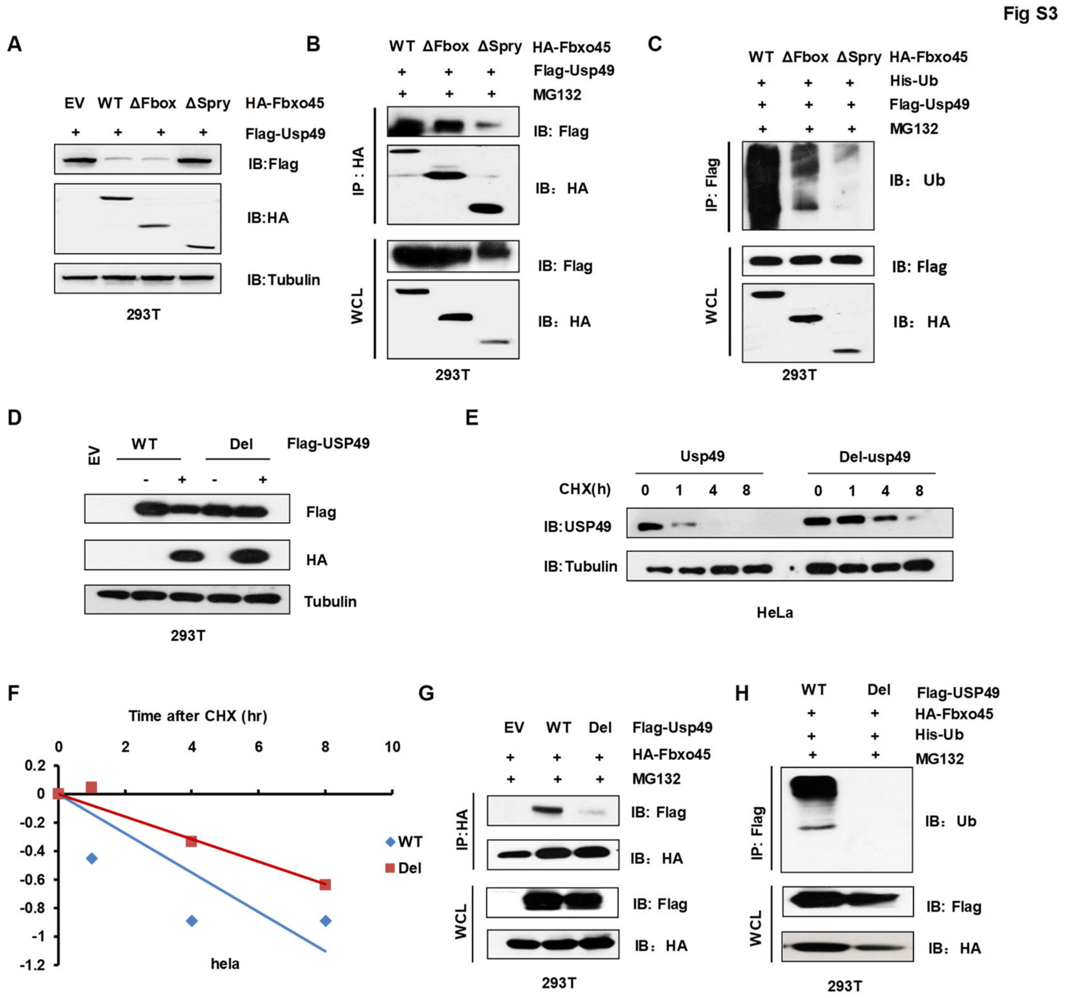

Figure S3: Fbxo45-induced ubiquitination and degradation of USP49 depends on its SPRY domain.

A. IB analysis of WCLs derived from 293T cells transfected with the indicated plasmids.

B. IB analysis of IPs and WCLs derived from 293T cells transfected with the indicated plasmids. Cells were treated with 10 μM MG132 for 6 hours before harvesting.

C. IB analysis of ubiquitination products and WCLs derived from 293T cells transfected with the indicated constructs. Cells were treated with 10 μM MG132 for 6 hours before harvesting.

D. IB analysis of WCLs derived from 293T cells transfected with the indicated plasmids.

E. IB analysis of WCLs derived from HeLa cells after the specified duration of 100 μg/ml cycloheximide (CHX) transfection with the indicated constructs.

F. USP49 protein abundance in (E) was quantified and plotted.

G. IB analysis of IPs and WCLs derived from 293T cells transfected with the indicated plasmids. Cells were treated with 10 μM MG132 for 6 hours before harvesting.

H. IB analysis of ubiquitination products and WCLs derived from 293T cells transfected with the indicated constructs. Cells were treated with 10 μM MG132 for 6 hours before harvesting.

Fig S4

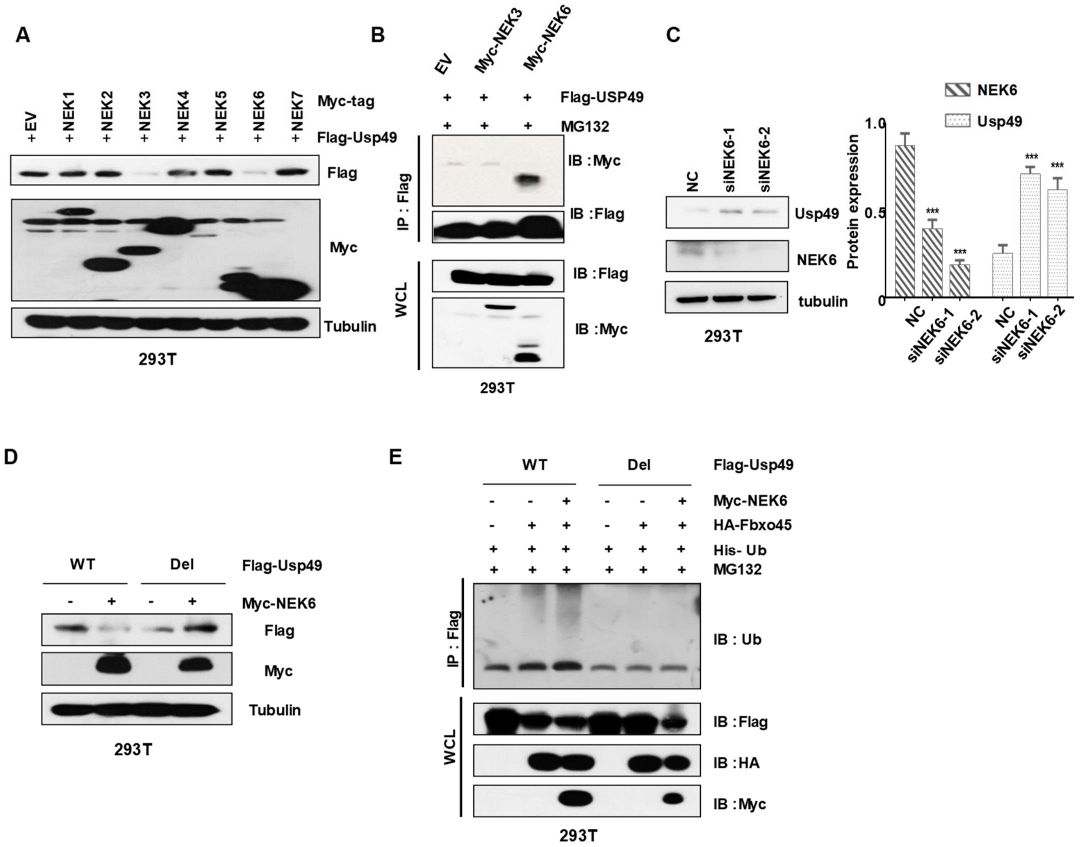

Figure S4: NEK6 enhances the degradation of USP49.

A. IB analysis of WCLs derived from 293T cells transfected with the indicated plasmids.

B. IB analysis of IPs and WCLs derived from 293T cells transfected with the indicated constructs. Cells were treated with 10 μM MG132 for 6 hours before harvesting.

C. IB analysis of WCLs derived from 293T cells transfected with the indicated plasmids (Left). The quantitative data were shown for left panel (Right).

D. IB analysis of WCLs derived from 293T cells transfected with the indicated plasmids.

E. IB analysis of IPs and WCLs derived from 293T cells transfected with the indicated constructs. Cells were treated with 10 μM MG132 for 6 hours before harvesting.

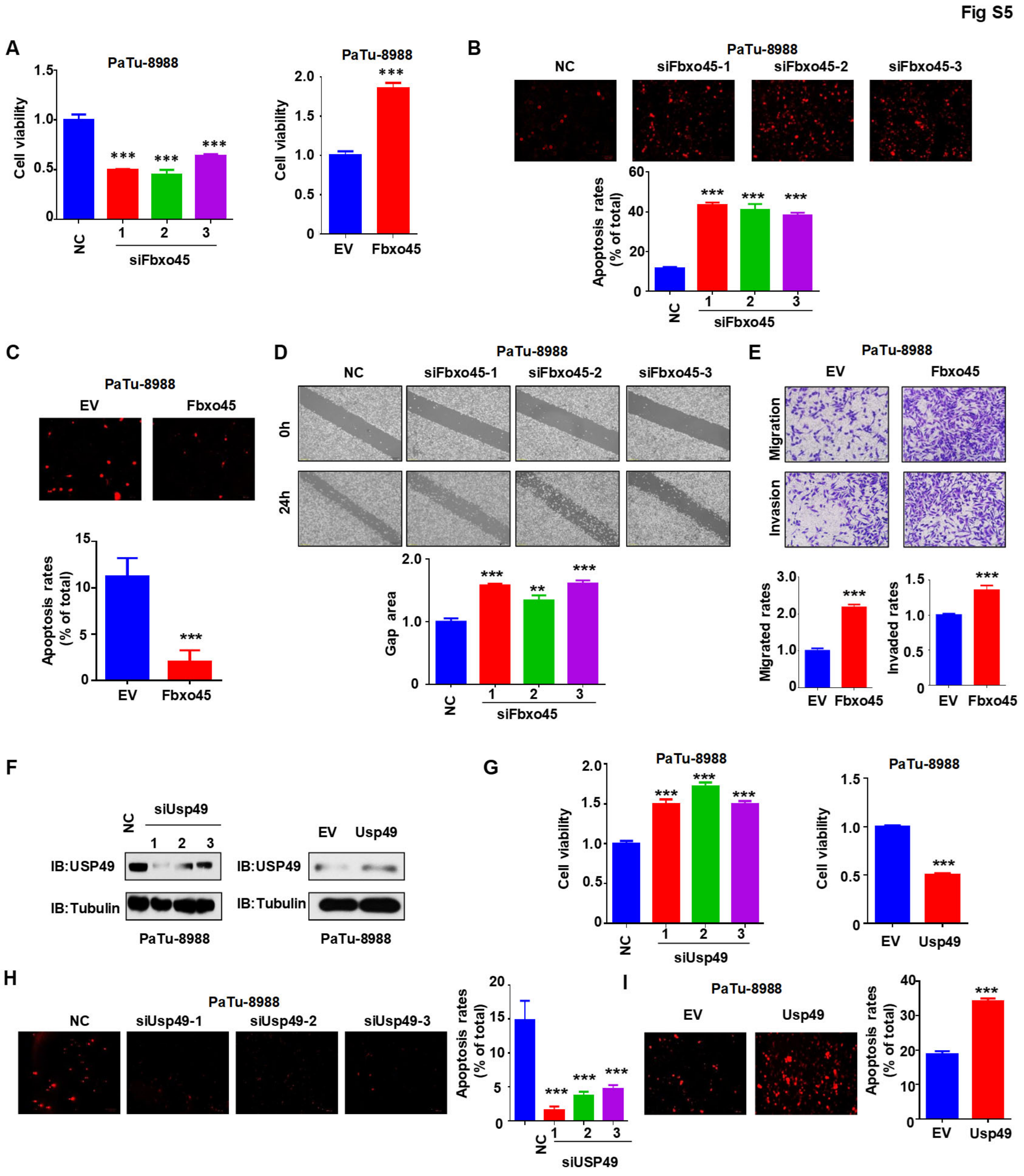

Figure S5. Fbxo45 promotes cell growth, migration and invasion in PC cells.

A. MTT assays to detect cell proliferation in PaTu-8988 cells transfected with Fbxo45 siRNAs (Left) or Fbxo45 constructs (Right). Data are shown as mean ± SD of three independent experiments. ***p<0.001 compared to control.

B-C. TUNEL assays to detect cell apoptosis of PaTu-8988 cells transfected with Fbxo45 siRNAs (B) or Fbxo45 constructs (C).

D. Wound healing assays to analyze cell migration capacity of PaTu-8988 cells transfected with Fbxo45 siRNAs. **p<0.01, ***p<0.001 compared to control.

E. Transwell assays to analyze cell migration and invasion capacity of PaTu-8988 cells transfected with Fbxo45 cDNA. ***p<0.001 compared to control.

F: IB analysis of WCLs derived from PaTu-8988 cells transfected with the indicated plasmids.

G: MTT assays to detect cell proliferation in PaTu-8988 cells transfected with USP49

siRNAs (Left) or USP49 constructs (Right). Data are shown as mean ± SD of three independent experiments. ***p<0.001 compared to control.

H-I. TUNEL assays to detect cell apoptosis of PaTu-8988 cells transfected with USP49 siRNAs (H) or USP49 constructs (I). ***p<0.001 compared to control.

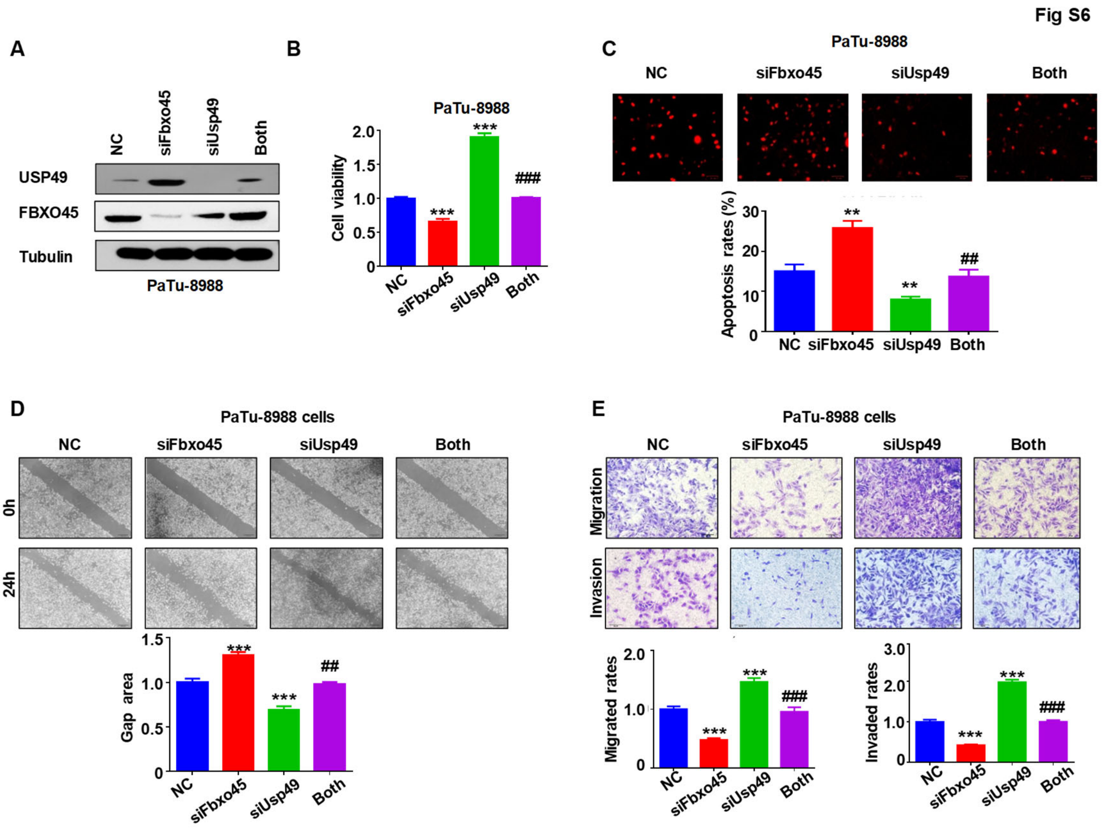

Figure S6: Fbxo45 negatively regulates USP49-mediated cell proliferation, migration and invasion.

A. IB analysis of WCLs derived from PaTu-8988 cells transfected with the indicated plasmids.

B. MTT assays to detect the proliferation of PaTu-8988 cells transfected with the indicated siRNAs. Data are shown as the mean ± SD of three independent experiments. ***p<0.001 compared to control, ### p<0.001 compared to Fbxo45 siRNA-2 alone or USP49 siRNA-2 alone.

C. TUNEL assays to detect cell apoptosis of PaTu-8988 cells transfected with the indicated siRNAs. **p<0.01 compared to the control, ## p<0.01 compared to Fbxo45 siRNA-2 alone or USP49 siRNA-2 alone.

D. Wound healing assays to analyze the cell migratory capacity of PaTu-8988 cells transfected with the indicated siRNAs. ***p<0.001 compared to control, ## p<0.01 compared to Fbxo45 siRNA-2 alone or USP49 siRNA-2 alone.

E. Transwell assays to analyze the cell migration and invasion capacity of PaTu-8988 cells transfected with the indicated siRNAs. ***p<0.001 compared to control, ### p<0.01 compared to Fbxo45 siRNA-2 alone or USP49 siRNA-2 alone.

Fig S7

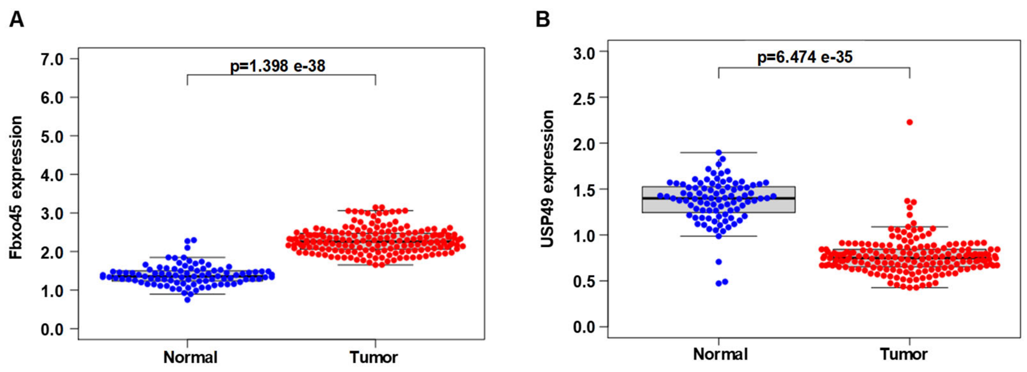

Figure S7: Fbxo45 expression is negatively associated with USP49 exprssion.
A-B: The bioinformatics results showed obviously higher expression of Fbxo45 and lower expression of USP49 in cancer tissues compared with normal tissues.
