# Supplementary material for: Fbxo45 facilitates pancreatic carcinoma progression by targeting USP49 for ubiquitination and degradation
Source: Cell Death Dis. 2022 Mar 12;13(3):231. doi: 10.1038/s41419-022-04675-2 (PMC8918322; doi:10.1038/s41419-022-04675-2)
Supplement: Supplementary file 3 — Editing certificate [file 41419_2022_4675_MOESM3_ESM.pdf]

Editing Certificate

This document certifies that the manuscript

Fbxo45 facilitates pancreatic carcinoma progression via targeting USP49 for ubiquitination and degradation

prepared by the authors

Linhui Wu, Ke Yu, Kai Chen, Xuelian Zhu, Zheng Yang, Qi Wang, Junjie Gao, Yingying Wang, Tong Cao, Hui Xu, Xueshan Pan, Lixia Wang，Jun Xia, Yuyun Li, Peter Wang, Jia Ma

was edited for proper English language, grammar, punctuation, spelling, and overall style by one or more of the highly qualified native English speaking editors at AJE.

This certificate was issued on **January 5, 2022** and may be verified on the AJE website using the verification code **3F30-DAD3-B93C-3E19-358F**.

Neither the research content nor the authors' intentions were altered in any way during the editing process. Documents receiving this certification should be English-ready for publication; however, the author has the ability to accept or reject our suggestions and changes. To verify the final AJE edited version, please visit our verification page at aje.com/certificate. If you have any questions or concerns about this edited document, please contact AJE at support@aje.com.

AJE provides a range of editing, translation, and manuscript services for researchers and publishers around the world. For more information about our company, services, and partner discounts, please visit aje.com.
